# Effective modulation from the ventral medial to the dorsal medial portion of the prefrontal cortex in memory confidence-based behavioral control

**DOI:** 10.1038/s41598-024-60755-7

**Published:** 2024-05-02

**Authors:** Shoko Yuki, Hironori Nakatani, Ryosuke O. Tachibana, Kazuo Okanoya

**Affiliations:** 1 Graduate School of Arts and Sciences, The University of Tokyo, 3-8-1, Komaba, Meguro-ku, Tokyo, 153-8902 Japan; 2 School of Information and Telecommunication Engineering, Tokai University, 2-3-23, Minato-ku, Takanawa, Tokyo, 108-8619 Japan; 3 Advanced Comprehensive Research Organization, Teikyo University, 2-21-1, Kaga, Itabashi-ku, Tokyo, 173-0003 Japan

**Keywords:** Cognitive neuroscience, Human behaviour

## Abstract

Metacognition includes the ability to refer to one’s own cognitive states, such as confidence, and adaptively control behavior based on this information. This ability is thought to allow us to predictably control our behavior without external feedback, for example, even before we take action. Many studies have suggested that metacognition requires a brain-wide network of multiple brain regions. However, the modulation of effective connectivity within this network during metacognitive tasks remains unclear. This study focused on medial prefrontal regions, which have recently been suggested to be particularly involved in metacognition. We examined whether modulation of effective connectivity specific to metacognitive behavioral control is observed using model-based network analysis and dynamic causal modeling (DCM). The results showed that negative modulation from the ventral medial prefrontal cortex to the dorsal medial prefrontal cortex was observed in situations that required metacognitive behavioral control but not in situations that did not require such metacognitive control. Furthermore, this modulation was particularly pronounced in the group of participants who could better use metacognition for behavioral control. These results imply hierarchical properties of metacognition-related brain networks.

## Introduction

When we forget the correct route, we check the map to confirm it and start walking. This ability to control behavior according to the certainty of one’s memory is a form of metacognition^1,2^. These prospective and voluntary behavioral control mechanisms help us prevent future problems (e.g., getting lost). Metacognition is thought to be realized by a twofold system of cognition that processes stimuli from the external world (object level) and monitors and controls them (meta-level)^3^. Regarding its neural implementation, Shimamura^4,5^ has proposed the “Dynamic Filtering Theory (DFT)” from the earliest years of the study of the neural basis of metacognition. This theory hypothesizes that object-level processes are distributed in the cortical regions posterior to the prefrontal cortex, which are monitored and controlled by meta-level processes in the prefrontal cortex. Here, the prefrontal cortex is considered hierarchically upstream from other posterior regions and is responsible for enabling proper decision-making by enhancing appropriate signals, suppressing inappropriate signals, and sending them back to the posterior regions.

Regarding the location of the region responsible for metacognition, functional magnetic resonance imaging (fMRI) studies began to report results around 2010, and early results suggested that the lateral prefrontal cortex was the site of the meta-level process^6,7^. Subsequently, findings have emerged suggesting the involvement of the medial prefrontal cortex, particularly concerning metacognition for memory (metamemory), as individual differences in metamemory performance correlate with functional connectivity between the anterior medial prefrontal cortexes (amPFC) and the precuneus or inferior parietal lobule (IPL)^8^. Vaccaro and Fleming^9^ conducted a meta-analysis of 47 neurophysiological studies on metacognition, which suggested that the lateral prefrontal cortex, as well as the ventral and dorsal medial prefrontal cortex, is involved in meta-level processing, regardless of whether the target domain is perception or memory. Noteworthy, the analysis included studies on feelings of knowing and judgments of learning and not only on confidence in one’s own decisions. This suggests that these brain regions are broadly involved in metacognition as judgments about one’s own cognitive process and not just confidence. Furthermore, it has been reported that the activity of the ventral medial prefrontal cortex specifically corresponds to both within-subject and between-subject variability in estimated confidence in perceptual tasks^10^. Thus, the medial prefrontal cortical regions (especially the ventral part), rather than the lateral prefrontal cortex, may play a considerable role in meta-level processing.

Therefore, examining changes in activity in the ventral and dorsal medial prefrontal cortices during metacognitive processing should contribute to the elucidation of the neural mechanism of metacognition. Since each region within the medial prefrontal cortex has reciprocal connectivity^11^, these regions are expected to be involved in metacognition by interacting as a network rather than individually. However, modulations in effective (directional) connectivity among regions during metacognition are still not understood. In general, the dorsal part of the medial prefrontal cortex is thought to be involved in cognitive control^12^, such as increasing activity in response to the degree of conflict among multiple competing options^13^. In contrast, the ventral part is thought to be involved in self-referential processes^14^, such as information about the self, subjective values, and emotions^15^. About metacognition, it has been suggested that the ventral medial PFC (vmPFC) is specifically involved in metacognitive monitoring^10^, whereas the dorsal medial (dmPFC) is specifically involved in metacognitive control^16^. Thus, metacognitive processing predicts that the information about one’s cognitive processes expressed in the vmPFC is propagated to the dmPFC and sequentially to the regions responsible for actual behavioral output.

The present study focused on confidence as a component of the metacognitive processes. It tested this hypothesis by performing a model-based analysis of modulations in effective connectivity between medial prefrontal regions during confidence-based memory bet selection using blood oxygenation level-dependent (BOLD) signal data from our previous fMRI study^17^.

## Methods

### Summary of the experiments in Yuki et al***.***^17^

**Figure 1:**
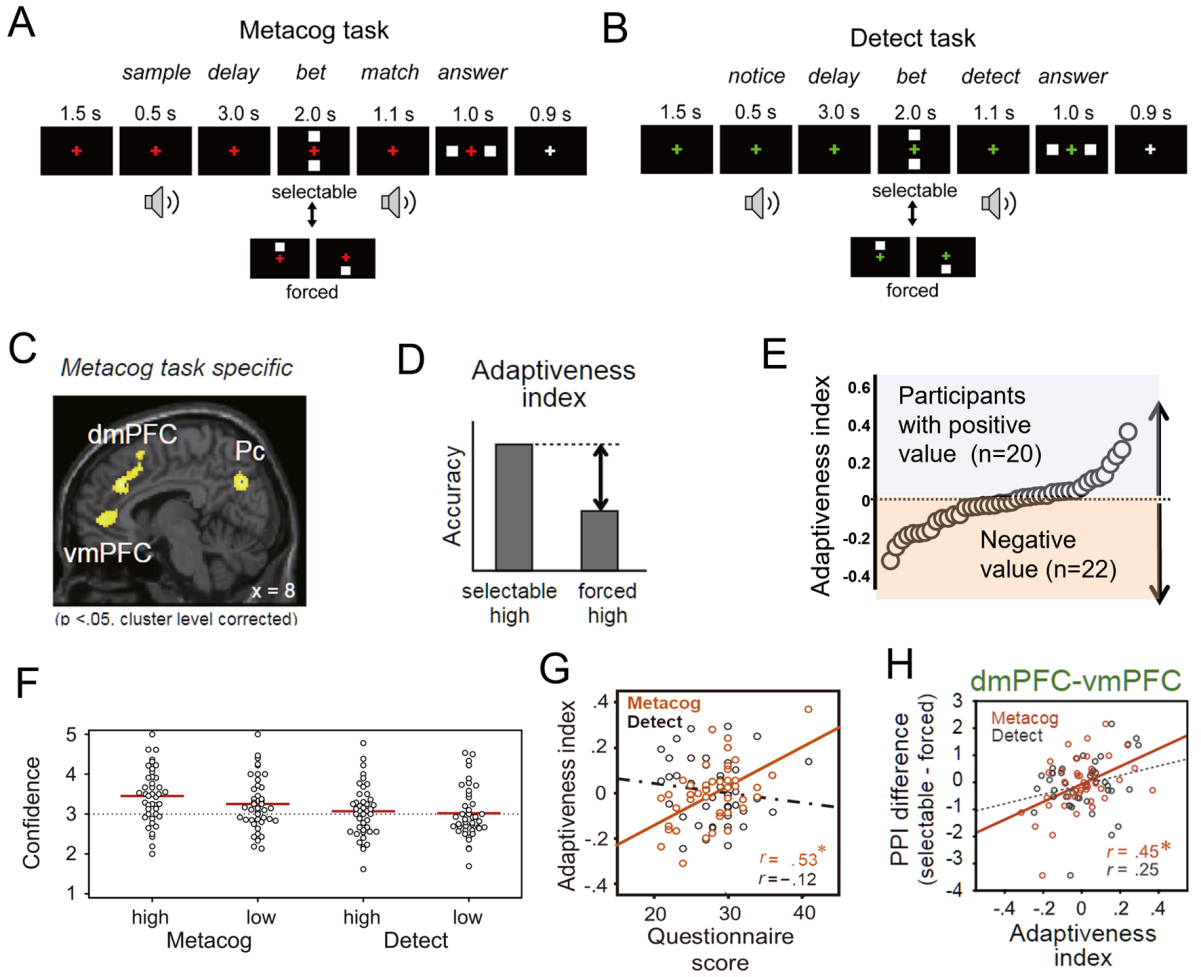
Schematic of the screens presented to participants in the task used in the experiment (**A**,**B**). Time sequence of Metacog (**A**) and Detect (**B**) trials, with fixation crosses in red and green indicating the Metacog and Detect task, respectively. The white squares presented above and below the fixation cross in the betting phase corresponded to the risk/reward that participants could take. The upper square corresponded to high-risk/high-return. The lower square corresponded to low-risk/low-return. (**C**) Dorsal and ventral medial prefrontal cortices (dmPFC and vmPFC, respectively), as well as the medial precuneus region (Pc), showed increased activity during bet selections in the Metacog task compared with when forced to choose one or the other, but no change in activity in an equivalent comparison in the Detect task. (**D**) Definition of the adaptiveness index. (**E**) The averages of the adaptiveness index for each participant in ascending order. One white circle corresponds to one participant. (**F)** Average confidence score for the selected high-risk/high-return and low-risk/low-return trials in the Metacog and Detect tasks. Red bars show the mean value across participants. (**G**) Interindividual correlations between the adaptiveness index in the Metacog and Detect tasks and scores on a questionnaire on the use of prospective metacognition in everyday life. (**H**) Interindividual correlation of the adaptiveness index and similar condition difference for vmPFC-dmPFC synchronization (PPI; psychophysiological interaction). (**A–D**,**F–H)** were adapted from Yuki et al*.*^17^. (**E)** was adapted with modification from Yuki et al*.*^26^^.^

The following is a summary of the experiments and analyses performed by Yuki et al*.*^17^. Forty-two participants (20 women; age range 18–23 years, mean ± SD 19.4 ± 1.0 years) engaged in a delayed matched-to-sample task as a *Metacog task* where they listened to a brief sound stimulus that differed with each trial, listened to a second sound 3 s after the first one, and then differentiated between the first and second sounds (Fig. 1A). In half of the trials, before listening to the second sound stimulus, participants were required to bet on either a high-risk/high-return or low-risk/low-return selection, depending on their prediction about whether they would answer correctly in that trial. Based on the bet selection and the correctness of the answer, the participants gained or lost points in each trial. The participants were instructed to maximize their total score per session, but the experimental remuneration was fixed and independent of their scores.

The only valid cue that participants could rely on to predict the correctness of their answers at the time of the bet was the memory confidence of the first sound stimulus that they had heard 3 s earlier. Therefore, it can be assumed that participants who selected the high-risk/high-return option in trials that could be answered correctly based their bets on their memory confidence. Participants were presented with only one of the two bet options in the remaining half of the trials. In addition, the same participants also performed a control task called the *Detect task* that had the same stimulus presentation timing and required the same bet selection. However, there was no need to remember details of the first sound stimulus for the bet (Fig. 1B). In this task, participants had to discriminate whether the sound presented at the time of the second sound stimulus was only noise or contained a tone. The first stimulus served only as a preview of the upcoming noise level, providing information about the task’s difficulty. Therefore, the participants did not have to remember the tone itself.

We identified regions that showed significantly increased activity during the betting phase in the Metacog task in the condition in which participants could select their own bets compared with the condition in which they could only take one of the betting options. To eliminate the influence of out-of-interest changes in brain activity related to the bet selection, such as motivation for the experiment and risk preference, regions that showed significant increases in the same contrast subtraction of the Detect task were excluded from this analysis by exclusive masking. In particular, risk preference could have strongly influenced participants’ bet selections during the experiment. However, in the original experiment, the rates of high-risk/high-return selections did not significantly differ across tasks. Thus, the above masks are expected to remove this influence. See Sect. 3.1.4 of^17^ for further details. We found significantly higher activation in the ventral (peaks at x = −2, y = 42, z = 6 mm in MNI coordinates; vmPFC) and dorsal (8, 34, 32; dmPFC) regions of the medial prefrontal cortex and precuneus (−4, −70.34; Pc); thus, these three regions were considered responsible for memory confidence-based bet selection (Fig. 1C).

In addition to these three regions, brain activity in the left visual cortex (−22, −98, −2; VA) showed similar significant condition differences but was excluded from further analysis. This was because the significant differences were thought to be due to differences in the images displayed on the bet screen in the selectable and forced conditions. This reason was supported by an additional analysis that found that the left and right visual cortices increased their activities in the selectable rather than the forced condition common to the two tasks.

The “adaptiveness index” was defined as a behavioral index reflecting the success of confidence-based bet selections in the Metacog task. This index was calculated by subtracting the accuracy when the participants were forced to take a high-risk/high-return option from that when they dared to select a high-risk/high-return option (Fig. 1D). A larger value of this index means that participants selected the high-risk/high-return option in trials that could be answered correctly. In other words, it is assumed that in the Metacog task, the adaptiveness index reflects the extent to which the bets relied on memory confidence. As shown in Fig. 1E, large individual differences in adaptiveness index values were observed among the participants. A behavioral experiment preceded the brain activity measurements, and the confidence was retrospectively reported on a 5-point Likert scale, confirming the consistency of the betting options selected in each trial (Fig. 1F). Furthermore, participants with high adaptiveness index scores in Metacog task tended to score higher on questions related to prospective metacognition, such as planning, goal setting, and resource allocation, prior to learning. However, this correlation was not found in the control task (Fig. 1G). These results suggest that participants selected bets based on their memory confidence in Metacog task, although approximately half of them did not take advantage of the opportunity to select their own bets. Individual differences in the adaptiveness index were significantly correlated with the same between-condition differences in the functional connectivity of the vmPFC and dmPFC (i.e., their degree of synchronization; Fig. 1H).

In summary, Yuki et al*.*^17^ have shown that the activity levels in the vmPFC and dmPFC change during confidence-based bet selection and that the functional connectivity between these regions is significantly correlated with the adaptiveness index of participants, but they did not examine the effective connectivity between medial prefrontal cortex regions. For further details, please refer to the original publication.

### Dynamic causal modeling (DCM) analysis

To investigate the modulation of effective connectivity within the medial prefrontal cortex regions during metacognitive processing, we conducted a model-based analysis using the DCM method^18^, implemented in SPM8 (Welcome Trust Centre for Neuroimaging, University College London, UK). DCM estimates the intrinsic effective connectivity among brain regions and how much this connectivity is modulated by experimental manipulations (e.g., stimulus presentations) based on the temporal dynamics of BOLD signals. This analysis separately estimates the intrinsic effective connectivity or its modulation from region A to region B and vice versa.

The coefficients obtained from the DCM analysis are estimated degrees of how much the modeled neuronal states in region A at time point t can explain the neuronal states in region B at the next time point t + 1. Thus, a positive/negative coefficient indicates a relationship where an increased activity in region A leads to an increased/decreased activity, respectively, in region B.

The procedure for DCM analysis is as follows. First, the experimenter builds a model based on hypotheses about which regions may have intrinsic connectivity and change their activity in response to experimental manipulations and which connectivity is affected by such inputs. Second, the coefficients for the intrinsic connectivity, its modulations, and the inputs causing the modulation were calculated by fitting the actual experimental data to the constructed model. Therefore, as the number and/or direction of propagation paths in a model change, the estimated coefficients for the same paths, as well as the fitness of the data, will change across models. To avoid bias in the results due to the hypotheses, we prepared several models with different propagation pathways, applied them to the same experimental data, compared their fitness, and searched for the best propagation model.

### Model search 1: the networks associated with metacognition

We first searched for the best-fitting model among all participants to examine the modulation of effective connectivity in the network when confidence-based behavioral control was required.

#### Model specification

We defined three-dimensional regions of interest [volumes of interest (VOIs)] in a sphere with a radius of 8 mm from the abovementioned peak coordinates of the four regions (vmPFC, dmPFC, Pc, and VA) that showed in Yuki et al.^17^ specific activity changes for memory confidence-based bet selection and examined how effective connectivity among these regions is altered by the presentation of bet-selectable screens. Since the trials were presented in random order and the participants did not know whether the bet options were selectable until the bet screen was presented, the visual input of the presented bet screen was thought to alter the effective connectivity.

We constructed two individual-level generalized linear models, including the following independent variables: timing of all bet selection (or forced) phases in the two conditions of the two tasks (for input), timing of only bet selection events in the selectable condition of the Metacog or Detect task (for modulation in effective connectivity), and dummy variables for the sessions to capture differences across sessions. It is assumed that the presentation of a bet screen in the two task conditions evokes activity in the visual cortex but only modulates effective connectivity among the four VOIs and autocorrelations within areas in conditions where participants could select bet options in either the Metacog or Detect task. The only difference between the two models was whether the independent variable corresponding to the factor that caused modulations in effective connectivity was the presentation of the bet-selectable screen in the Metacog task or that in the Detect task; otherwise, the two models were identical.

#### Procedures for optimal model search

We performed backward model selection in the search for the optimal model. This method first defined the full model and then reduced the number of pathways to make them more applicable to the data to obtain the optimal model. The full model was designed so that all possible combinations between two of the four VOIs could have a bidirectional effective connectivity relationship (a total of 16 pathways, including autocorrelation). For the analysis, the SPM8 function spm_dcm_post_hoc.m for DCM was used^19^.

When selecting the model, we sought the one that best fitted all 42 participants. The coefficients for each of the final remaining pathways were tested using the one-sample *t*-test to assess whether they differed significantly from 0 across participants when corrected for multiple comparisons using the false discovery rate (FDR) method^20^. Corrections for multiple comparisons were made separately for tests of intrinsic connectivity and for tests of input and the resulting modulations in connectivity between brain regions.

### Model search 2: the networks that ensure successful metacognition

As shown in Fig. 1E, there were large individual differences in the adaptiveness index. If the modulations of effective connectivity specific to confidence-based bet selection estimated in Model search 1 are also related to whether the confidence-based bet selection operates successfully, these modulations should be more significant for those who successfully performed confidence-based bet selections (index > 0) than for those who did not (index < 0). To test this prediction, we conducted Model search 2. Model search 2 was the same as Model search 1, except that participants were divided into two groups based on the positive (n = 20) and negative (n = 22) values of the adaptiveness index, and the best-fitting model for each group was selected individually.

## Results

### Model search 1: the networks associated with metacognition

**Figure 2:**
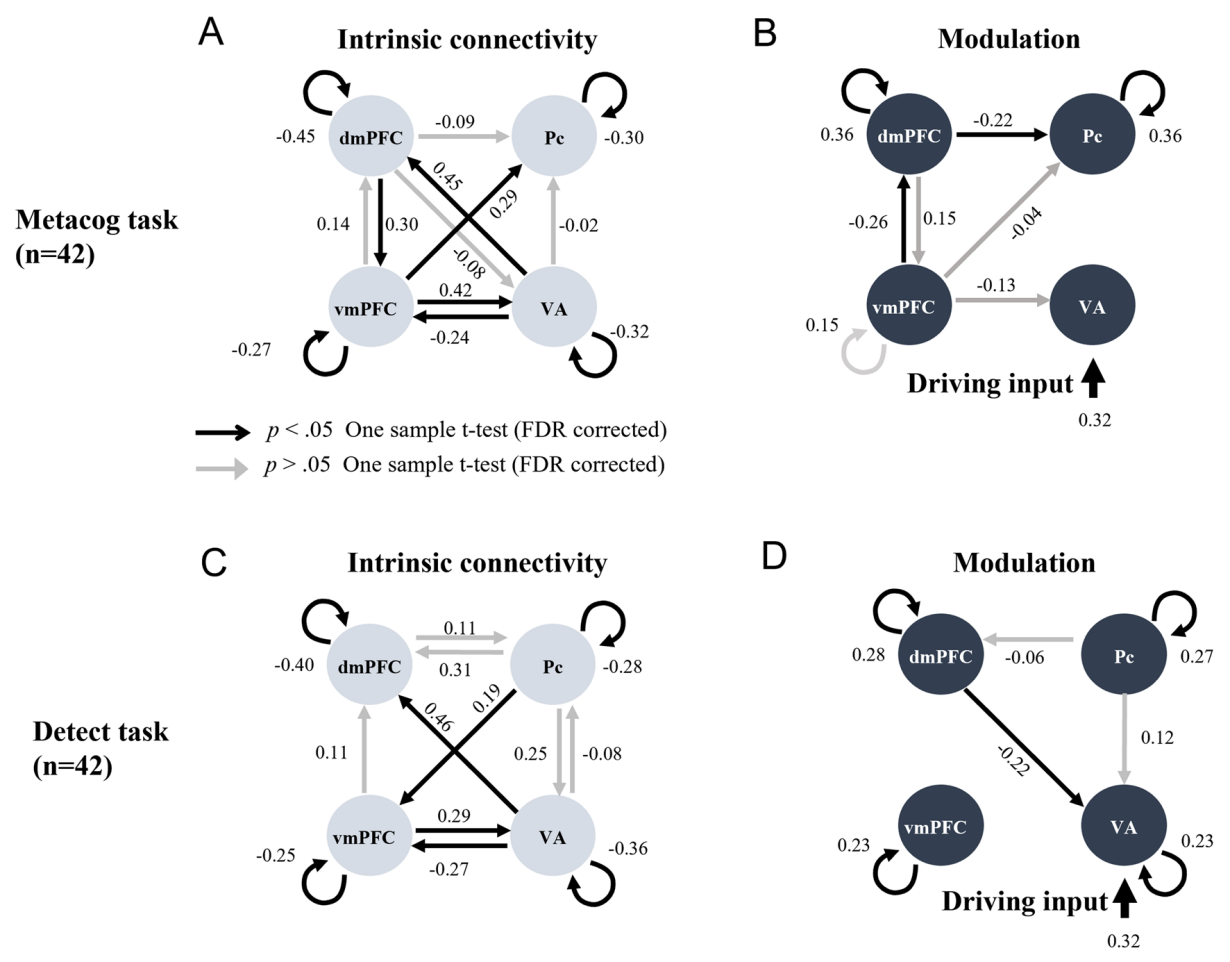
Intrinsic connectivity and modulation of the connectivity by the presentation of the bet-selectable screen of the Metacog (upper) or Detect (lower) task in the best model of Model search 1. The direction of the arrow indicates the direction of effective connectivity. Black arrows indicate effective connectivity paths included in the final model that were significant after correction for multiple comparisons (*p* < .05), whereas gray arrows indicate those that were not (*p* > .05). *vmPFC* ventral medial prefrontal cortex, *dmPFC* dorsal medial prefrontal cortex, *Pc* precuneus, *VA* visual area.

**Figure 3:**
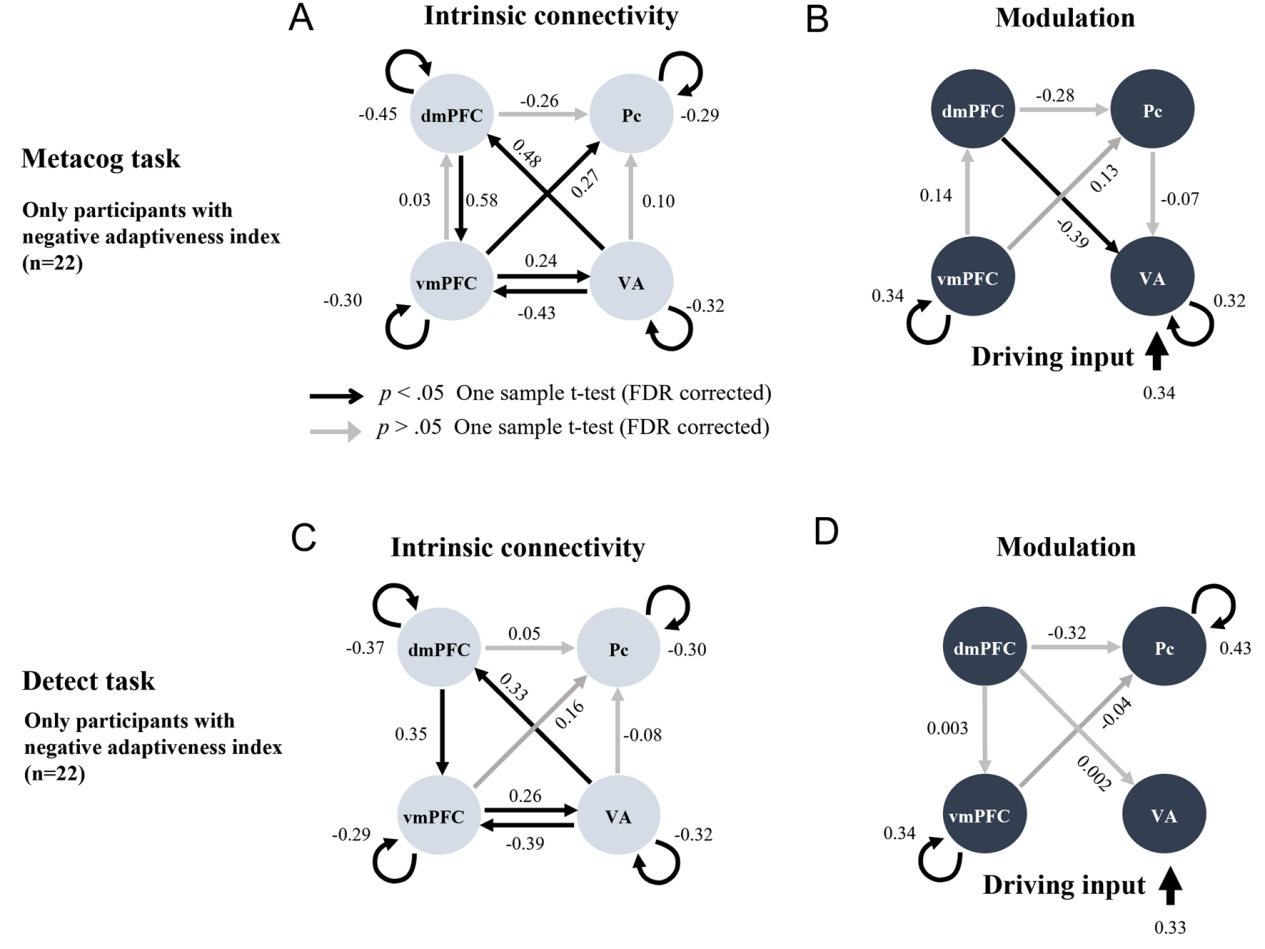
Intrinsic connectivity and modulation of the connectivity by the presentation of the bet-selectable screen of the Metacog (upper) or Detect (lower) task in the best model of Model search 2 only for participants with negative adaptiveness index values. The direction of the arrow indicates the direction of effective connectivity. Black arrows indicate effective connectivity paths included in the final model that were significant after correction for multiple comparisons (*p* < .05), whereas gray arrows indicate those that were not comparisons (*p* > .05).

**Figure 4:**
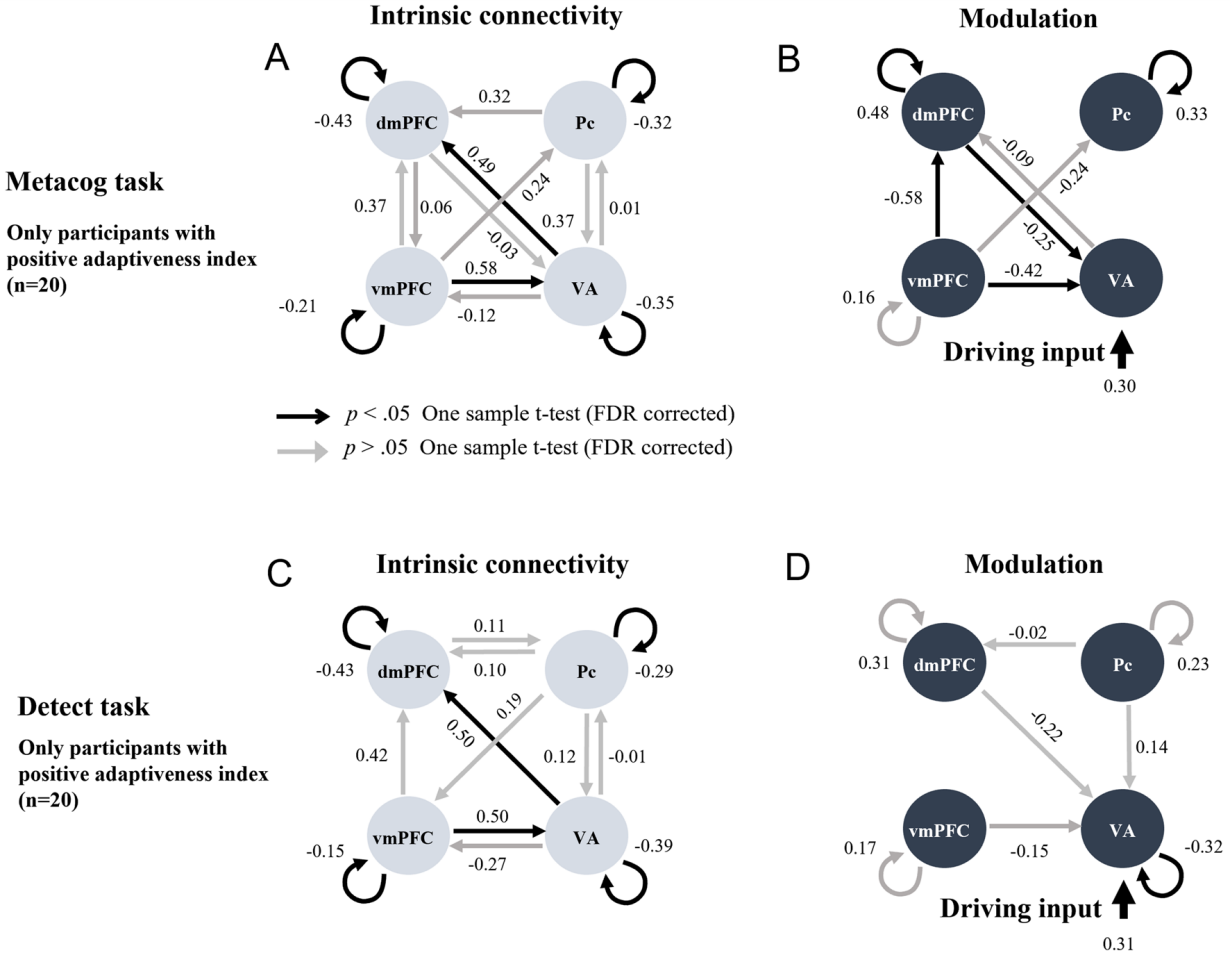
Intrinsic connectivity and modulation of the connectivity by the presentation of the bet-selectable screen of the Metacog (upper) or Detect (lower) task in the best model of Model search 2 only for participants with positive adaptiveness index values. The direction of the arrow indicates the direction of effective connectivity. Black arrows indicate effective connectivity paths included in the final model that were significant after correction for multiple comparisons (*p* < .05), whereas gray arrows indicate those that were not comparisons (*p* > .05).

In Figs. 2, 3 and 4, arrows indicate the direction of the estimated effective connectivity. Black arrows indicate significant paths after correction for multiple comparisons (significance level 0.05), whereas gray arrows indicate those that were not. The left side of the upper half of Fig. 2 shows the average coefficient of the estimated intrinsic connectivity, independent of the presentation of the bet-selectable screen. The coefficient value represents the percentage of the estimated relationship indicating that the activity at a given time t in the region where the arrow originates influences the activity at time t + 1 in the region where the arrow ends, not considering the influence from other regions. For example, if the coefficient from region A to region B is 0.3, the estimated effective connectivity is such that 30% of the activity in region A is added to the activity in region B. The average coefficient of modulation in effective connectivity resulting from bet selection in the Metacog task is shown on the Fig. 2B. In addition, the *t*-values of the one-sample *t*-test for all pathways remaining in the best model and their *p*-values after multiple comparisons are shown in the upper half of Table 1.

**Table 1:**
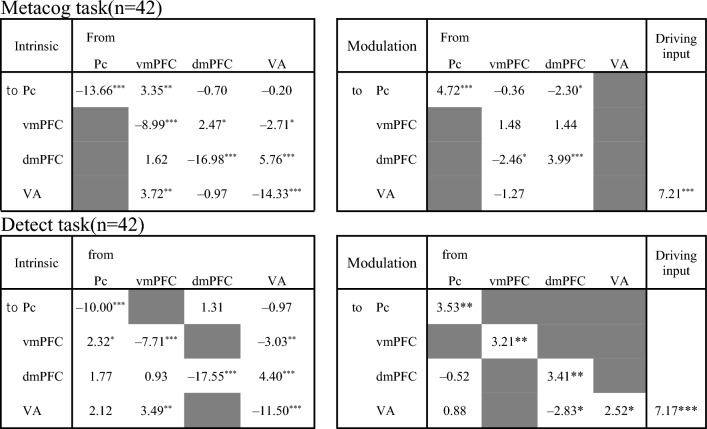
Statistics for the one-sample *t*-test performed on Model search 1.

The upper and lower sections correspond to Fig. 2. The values in each cell are the *t*-values of the one-sample *t*-test for each pathway (*df* = 41, *** *p* < .001, ** *p* < .01, * *p* < .05 corrected for multiple comparisons with the FDR method).

In terms of modulations in effective connectivity within the medial prefrontal cortex regions, consistently across participants, the presentation of bet-selectable screens in the Metacog task suggested to cause a negative modulation in effective connectivity from the vmPFC to the dmPFC and from the dmPFC to the Pc. By contrast, such modulations were not observed when bet-selectable screens were presented in the Detect task (Fig. 2D and lower half of Table 1). These results suggest that the presentation of the bet-selectable screen itself modulates effective connectivity within the medial prefrontal cortex, but only in the Metacog task, it does cause a modulation in effective connectivity from the vmPFC to the dmPFC.

### Model search 2: the networks that ensure successful metacognition

Focusing on the effective connectivity within the medial prefrontal cortex regions, participants with a negative adaptiveness index were estimated to have positive intrinsic connectivity from the dmPFC to the vmPFC, and the presentation of bet-selectable screens of the Metacog task did not modulate this connectivity (Fig. 3A, B and upper half of Table 2). On the other hand, participants with a positive index value were estimated not to have this intrinsic connectivity, and it was estimated that the negative modulation of effective connectivity from the vmPFC to the dmPFC was caused by the presentation of bet-selectable screens in the Metacog task (Fig. 4A, B and upper half of Table 3). Even when the model assumption was modulated so that the presentation of the bet-selectable screen in the Detect task altered effective connectivity, similar group differences were found for intrinsic connectivity. However, no modulation in effective connectivity within the medial prefrontal cortex was found with the presentation of this screen (Figs. 3C, D and Fig. 4C, D and lower halves of Tables 2 and 3).

**Table 2:**
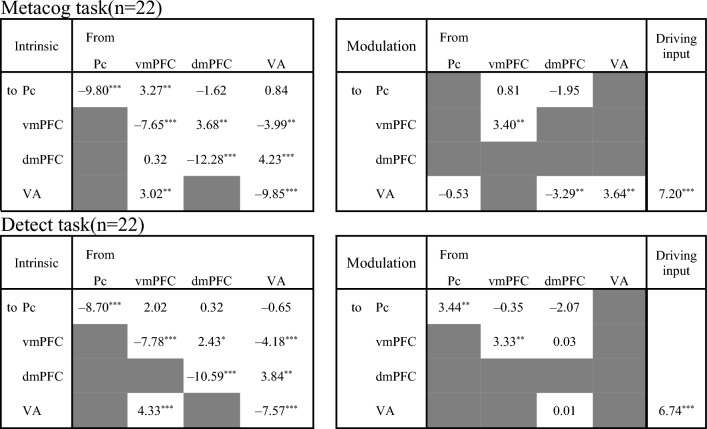
Statistics for the one-sample *t*-test performed on Model search 2 (only participants with negative adaptiveness index values).

The upper and lower sections correspond to Fig. 3. The values in each cell are the *t*-values of the one-sample *t*-test for each pathway (*df* = 21, *** *p* < .001, ** *p* < .01, * *p* < .05 corrected for multiple comparisons with the FDR method). Gray cells indicate that the pathway was dropped during model selection, and the final model did not assume considerable participation.

**Table 3:**
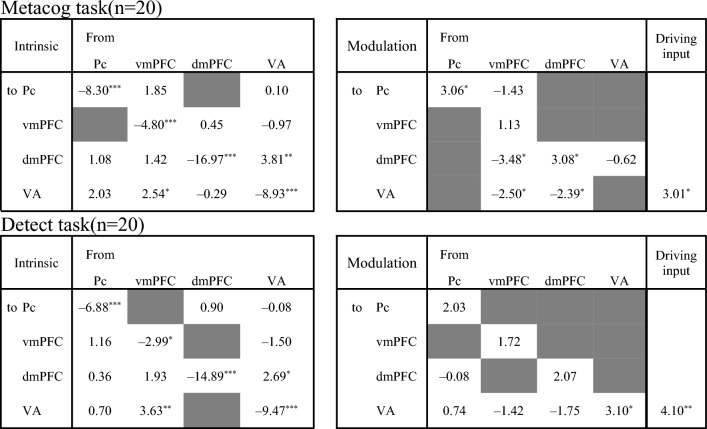
Statistics for the one-sample *t*-test performed on Model search 2 (only participants with positive adaptiveness index values).

The upper and lower sections correspond to Fig. 4. The values in each cell are the *t*-values of the one-sample *t*-test for each pathway (*df* = 19, *** *p* < .001, ** *p* < .01, * *p* < .05 corrected for multiple comparisons with the FDR method).

Focusing on the effective connectivity between the medial prefrontal cortex and other regions, there was a common positive intrinsic connectivity from the vmPFC to the VA and from the VA to the dmPFC, regardless of the adaptiveness index and tasks. Furthermore, negative intrinsic connectivity from the VA to the vmPFC was estimated only for participants with a negative index value, regardless of whether the presentation of the bet screen was assumed to modulate effective connectivity in the Metacog or Detect task.

## Discussion

This study aimed to examine the direction of effective connectivity within the medial prefrontal cortex related to confidence-based behavioral control as a form of metacognition. Therefore, we performed a DCM analysis to decompose and quantify the time-series changes in BOLD signals, which reflected brain activity into intrinsic connectivity and modulations in effective connectivity driven by specific experimental manipulations and assessed the effects of the presentation of scenes requiring bet selection based on memory confidence on effective connectivity within the brain regions of interest.

Within the medial prefrontal cortex, effective connectivity from the vmPFC to the dmPFC is negatively modulated during memory confidence-based bet selection. This negative modulation in effective connectivity suggests a relationship such that an increase (decrease) in activity in the vmPFC is associated with a subsequent decrease (increase) in activity in the dmPFC. This modulation was particularly strong in the group of participants who successfully performed the confidence-based bet selection. Considering these results and the functions of each brain region shown in previous studies^10,12,13^, it can be interpreted that the vmPFC represents the degree of memory confidence, and the dmPFC, which receives information, makes behavioral decisions. Possible interpretations of why a negative effective connectivity was observed are as follows. First, as mentioned in the Introduction, it has been suggested that vmPFC activity increases with higher confidence^10^, and similarly, dmPFC activity increases with a greater need for cognitive control^12^, such as greater conflict in choosing between multiple options^13^. In the present task, the higher the level of memory confidence, the clearer the advantage of the high-risk/high-return selection. Thus, there may have been less load in deciding whether to select one of the betting options.

The Pc, especially in the Metacog task, was almost always on the receiving end of information propagation to the prefrontal cortex regions. In other words, it was always lower in the hierarchy than the prefrontal cortex. This result is consistent with predictions based on the DFT hypothesis since it can be interpreted as indicating that the prefrontal cortex controls Pc activity in a higher-level hierarchy. This relationship of the Pc consistently receiving control from the medial prefrontal cortex is also consistent with reports of studies examining effective connectivity between brain regions related to the default mode network^21,22^.

Focusing on the effective connectivity between the medial prefrontal cortex and other regions, intrinsic positive effective connectivity was observed from the vmPFC to the VA and from the VA to the dmPFC, regardless of whether the participant successfully performed confidence-based bet selection. In addition to this innate connectivity, participants who successfully performed confidence-based bet selection showed negative connectivity modulation from the vmPFC to the VA and from the dmPFC to the VA when presented with the bet-selectable screen in the Metacog task. The medial prefrontal cortex had top-down control of visual cortex activity during metacognition. This finding is consistent with the suggestion from recent attentional research that the prefrontal cortex has top-down control over the sensory cortex during attention^23^. However, as summarized in^23^, studies on top-down control of visual attention in the prefrontal cortex often focus on the lateral prefrontal cortex and higher visual cortex. Therefore, further studies are needed to clarify the extent to which these findings apply to the functional interpretation of effective connectivity between the medial prefrontal cortex and the lower visual cortex found in the present study.

Participants who did not successfully perform confidence-based bet selections had negative connectivity modulations from the dmPFC to the VA during the presentation of the bet-selectable screen in the Metacog task but no modulation from their intrinsic vmPFC to VA connectivity. However, because they had intrinsic negative effective connectivity from the VA to the vmPFC, an intrinsic activity-balancing bidirectional network between the VA and vmPFC might have been established. It is interesting that the estimated intrinsic network differed between participants who successfully performed confidence-based bet selections and those who did not. This result suggests that differences in brain networks established daily can predict confidence-based behavioral control during a task.

As shown in Fig. 1E, there was a significant interindividual difference in the adaptiveness index, regardless of whether it was positive or negative. Previous studies have demonstrated similar interindividual variability^6,7^. It is already known that individual differences in metacognitive ability correspond with variations in brain structure^6^. Further research is needed to determine whether individual differences related to metacognition, such as the degree of success in confidence-based bet selection, correspond to brain structures in the regions examined in the current study and, if such a correspondence exists, whether such structural differences influence effective connectivity.

Some limitations of our research must be mentioned. First, it should be noted that participants, on average, did not take advantage of the opportunity to select their own bets based on their confidence in the Metacog task. This may initially seem strange but is not puzzling because the accuracy of confidence judgments depends on the timing of the judgment and is less accurate when made prospectively before responding to a discrimination task, as in this study, than when made retrospectively after responding to the task^24^. Furthermore, even the accuracy of retrospective confidence is often inferior to that of stimulus discrimination in the same task^25^. One factor contributing to the maladaptive bets might be that participants received a fixed amount of reward regardless of their task performance. Successful confidence-based bet selection might benefit from increasing experimental motivation by adapting the compensation based on task performance.

Second, the DCM analysis method is based on several assumptions inherent to the noninvasive estimation of effective connectivity^18^. It cannot be overemphasized that the results obtained in the present study are based on a model that focused only on four empirically determined areas and assumed that changes in activity and effective connectivity occur in these areas in accordance with the experimental hypothesis. The generality of our findings needs to be verified in future studies by testing hypotheses based on our results.

In summary, the DCM analysis suggests that the vmPFC, which regulates both dmPFC and VA activity, is involved in the top-down coupling when confidence-based behavioral control is successful. The function of the vmPFC is interpreted as the suppression of input information from the sensory cortex to the dmPFC and the transmission of memory confidence information to the dmPFC during bet selection. These findings will contribute to the elucidation of effective connectivity in brain networks related to metacognition, which has not been studied before.

## Data Availability

The datasets analyzed in the current study are available from the corresponding author upon request.

## References

[1] Flavell JH (1979). Metacognition and cognitive monitoring: A new area of cognitive-developmental inquiry. Am. Psychol..

[2] Flavell JH (1971). Stage-related properties of cognitive development. Cogn. Psychol..

[3] Nelson TO, Narens L (1990). Metamemory: A theoretical framework and new findings. Psychol. Learn. Motiv..

[4] Shimamura AP (2000). Toward a cognitive neuroscience of metacognition. Conscious. Cogn..

[5] Shimamura, A. P. A neurocognitive approach to metacognitive monitoring and control In *Handbook of Metamemory and Memory* (ed. Dunlosky, J. & Bjork, R. A.). 373–390 (Psychology Press, 2008).

[6] Fleming SM, Weil RS, Nagy Z, Dolan RJ, Rees G (2010). Relating introspective accuracy to individual differences in brain structure. Science.

[7] Fleming SM, Huijgen J, Dolan RJ (2012). Prefrontal contributions to metacognition in perceptual decision making. J. Neurosci..

[8] Baird B, Smallwood J, Gorgolewski KJ, Margulies DS (2013). Medial and lateral networks in anterior prefrontal cortex support metacognitive ability for memory and perception. J. Neurosci..

[9] Vaccaro AG, Fleming SM (2018). Thinking about thinking: A coordinate-based meta-analysis of neuroimaging studies of metacognitive judgements. Brain Neurosci. Adv..

[10] Bang D, Fleming SM (2018). Distinct encoding of decision confidence in human medial prefrontal cortex. Proc. Natl. Acad. Sci. USA.

[11] Haber SN, Behrens TE (2014). The neural network underlying incentive-based learning: Implications for interpreting circuit disruptions in psychiatric disorders. Neuron.

[12] Shenhav A, Cohen JD, Botvinick MM (2016). Dorsal anterior cingulate cortex and the value of control. Nat. Neurosci..

[13] Botvinick MM, Cohen JD, Carter CS (2004). Conflict monitoring and anterior cingulate cortex: An update. Trends Cogn. Sci..

[14] D’Argembeau A (2013). On the role of the ventromedial prefrontal cortex in self-processing: The valuation hypothesis. Front. Hum. Neurosci..

[15] Hiser J, Koenigs M (2018). The multifaceted role of the ventromedial prefrontal cortex in emotion, decision making, social cognition, and psychopathology. Biol. Psychiatry.

[16] Su J, Jia W, Wan X (2022). Task-specific neural representations of generalizable metacognitive control signals in the human dorsal anterior cingulate cortex. J. Neurosci..

[17] Yuki S, Nakatani H, Nakai T, Okanoya K, Tachibana RO (2019). Regulation of action selection based on metacognition in humans via a ventral and dorsal medial prefrontal cortical network. Cortex.

[18] Friston KJ, Harrison L, Penny W (2003). Dynamic causal modelling. Neuroimage.

[19] Friston K, Penny W (2011). Post hoc Bayesian model selection. Neuroimage.

[20] Benjamini, Y. & Hochberg, Y. Controlling the false discovery rate: A practical and powerful approach to multiple testing. *J. R. Stat. Soc. Ser. B Stat. Methodol.* **57**, 289–300 (1995).

[21] Di X, Biswal BB (2014). Identifying the default mode network structure using dynamic causal modeling on resting-state functional magnetic resonance imaging. Neuroimage.

[22] Jiao Q (2011). Granger causal influence predicts BOLD activity levels in the default mode network. Hum. Brain Mapp..

[23] Paneri S, Gregoriou GG (2017). Top-down control of visual attention by the prefrontal cortex: Functional specialization and long-range interactions. Front. Neurosci..

[24] Siedlecka M, Paulewicz B, Wierzchoń M (2016). But I was so sure! Metacognitive judgments are less accurate given prospectively than retrospectively. Front. Psychol..

[25] Shekhar M, Rahnev D (2021). Sources of metacognitive inefficiency. Trends Cogn. Sci..

[26] Yuki S, Sakurai Y, Okanoya K (2019). The utility of internal cognitive states as discriminative cues affecting behavioral adaptation in humans and animals. Anim. Behav. Cogn..

